# Association of Polymorphisms of the *CHI3L1* Gene with Asthma and Atopy: A Populations-Based Study of 6514 Danish Adults

**DOI:** 10.1371/journal.pone.0006106

**Published:** 2009-07-01

**Authors:** Camilla Noelle Rathcke, Johan Holmkvist, Lise Lotte N. Husmoen, Torben Hansen, Oluf Pedersen, Henrik Vestergaard, Allan Linneberg

**Affiliations:** 1 Department of Endocrinology, Herlev Hospital, University of Copenhagen, Copenhagen, Denmark; 2 Hagedorn Research Institute, Steno Diabetes Center, Gentofte, Denmark; 3 Research Centre for Prevention and Health, Glostrup Hospital, University of Copenhagen, Copenhagen, Denmark; 4 Faculty of Health Sciences, University of Southern Denmark, Odense, Denmark; 5 Faculty of Health Sciences, University of Aarhus, Aarhus, Denmark; 6 Faculty of Health Sciences, University of Copenhagen, Copenhagen, Denmark; LMU University of Munich, Germany

## Abstract

**Background:**

YKL-40 is a chitinase-like glycoprotein encoded by the chitinase 3-like 1 gene, *CHI3L1*, localized at chromosome 1q32.1. Increased levels of serum YKL-40 have been reported to be a biomarker for asthma and a reduced lung function. Interestingly, the C-allele of the -131 C→G (rs4950928) polymorphism of *CHI3L1* has been shown to associate with bronchial hyperresponsiveness and reduced lung function suggesting that variations in *CHI3L1* may influence risk of asthma. The objective of the present study was to investigate the association of common variation in the *CHI3L1* locus with asthma, atopy and lung function in a large population-based sample of adults.

**Methods/Principal Findings:**

Eleven single nucleotide polymorphisms (SNPs) of *CHI3L1* including rs4950928 were genotyped in 6514 individuals. Asthma was defined as self-reported history of physician-diagnosed asthma. Total IgE and specific IgE to inhalant allergens were measured on serum samples. Lung function was measured by spirometry. Homozygosity of the rs4950928 G allele as compared to homozygosity of the C allele was associated with self-reported physician diagnosed asthma (OR 1.5 (95% CI, 1.00–2.26)) and with prevalence of atopic asthma (OR 1.93 (95% CI, 1.21–3.07)) after adjustment for age, sex, smoking status, socio-economic class and BMI. Carriers of rs883125 G allele had a significantly lower prevalence of atopy (OR 0.82 (CI, 0.72; 0.94)) as compared to homozygosity of the C allele. None of the SNPs examined were significantly associated with FEV1. However, two SNPs (rs10399931and rs4950930) appeared to be significantly associated with FEV_1_/FVC-ratio. Subgroup analyses of never-smokers did not consistently influence the associations in an either positively og negatively way.

**Conclusions:**

In contrast to previous studies, the rs4950928 G allele, and not the C allele, was found to be associated with asthma. A few other SNPs of the *CHI3L1* was found to be significantly associated with atopy and FEV1/FVC ratio, respectively. Thus, more studies seem warranted to establish the role of *CHI3L1* gene in asthma and atopy.

## Introduction

YKL-40 is a chitinase-like glycoprotein encoded by the chitinase 3-like 1 gene, *CHI3L1*, localized at chromosome 1q32.1. YKL-40 belongs to the evolutionary conserved family of 18-glycosyl hydrolases, which consists of enzymatic active chitiniases as well as chitinase-like proteins without enzymatic properties. Chitinases function as effector molecules and mediate airway inflammation in both human epithelial cells [1] and in mouse models of asthma [2], [3]; however, despite the lack of enzymatic properties, YKL-40 levels are elevated in serum and in the lungs of patients with asthma where they correlate positively with the severity of the disease and inversely with lung function [4] indicating a participation of YKL-40 in the activity level and/or the pathogenesis of the disease.

A study investigating various polymorphisms of *CHI3L1* in relation to sarcoidosis has found that the *CHI3L1* -329 G/A polymorphism (rs10399931) contributes to interindividual variations of YKL-40 levels in patients with sarcoidosis but does not influence sarcoidosis susceptibility or disease severity [5]. In a genome wide association study *CHI3L1* was found to be a susceptibility gene for asthma, bronchial hyperresponsiveness and reduced lung function [6]. Furthermore, an elevated circulating YKL-40 level was a biomarker for asthma and reduced lung function [6]. In relation to asthma, the causal SNP was -131 C→G (rs4950928) in the core promoter region of *CHI3L1*. Recently, the -247C/T promoter polymorphism (rs10399805) of *CHI3L1* was found to associate with serum YKL-40 levels and the risk of atopy in children [7].

The objective of the present study was to investigate the putative association of common variation in the *CHI3L1* locus with asthma, atopy and asthma quantitative traits including measures of lung function in a large sample of adults Danes.

## Methods

### Ethics Statement

Informed written consent was obtained from all participants before participation. The study was approved by the Ethical Committees of Copenhagen and Aarhus and was in accordance with the principle of the Helsinki Declaration II.

### Study population

The current study is based on the baseline examination of participants in the Inter99 study, a population-based randomized controlled trial, investigating the effect of lifestyle intervention (smoking cessation, increased physical activity, and healthier dietary habits) on CVD [8]. The present study was focused on the baseline data before any intervention had been offered. Data were collected with self-administered questionnaires, a physical examination, and blood tests. Details on the study population, health examination, and the intervention program have been described elsewhere [8]. Briefly, the Inter99 study population were residents in the southern part of the former Copenhagen County. An age- and sex-stratified random sample of 13,016 men and women born in 1939–40, 1944–45, 1949–50, 1954–55, 1959–60, 1964–65, and 1969–70 was drawn from the Civil Registration System and invited to participate in a health examination during 1999–2001, so that they were aged 30, 35, 40, 45, 50, 55, 60, and 65 years on the day of the examination. A total of 12,934 were eligible for invitation. The baseline participation rate was 52.5% (n = 6,784). Only participants with a Northern European nationality were included in the current study (n = 6,514).

### Measurement of serum total IgE, serum specific IgE to aeroallergens and lung function

Measurement of specific IgE in serum is the gold standard for assessment of respiratory allergy in large-scale epidemiological studies. Serum samples collected at baseline were analysed for serum total IgE as well as serum specific IgE against four inhalant allergens (birch, grass, cat, and house dust mite (Dermatophagoides pteronyssinus)) by using the Immulite® 2000 Allergy Immunoassay System [9], [10]. The four allergens chosen reflects the most common and clinical important inhalant allergies in Denmark. A positive test to at least one of these four allergens will identify up to 90% of individuals skin prick test positive to at least one inhalant allergen in a standard panel of 10 inhalant allergens in the background population studied. Atopy was defined as a positive test for specific IgE (≥0.35 kU/L) to at least one of the four allergens.

Spirometry was performed by using the Cardiosoft® software (GE Medical Systems, Freiburg, Germany) and LF501 respiration flow transducer (Erich Jaeger B.V. and Marquette Hellige GmbH, Freiburg, Germany). Predicted values of FEV_1_ and FVC were calculated [11]. Measured values of FEV_1_ and FVC were expressed as per cent of predicted.

### Definition of asthma and atopic asthma

Self-reported physician-diagnosed asthma was defined as a confirmative answer to the question *“Has a physician ever told you that you have asthma?”*

Atopic asthma was defined as self-reported physician-diagnosed asthma combined with atopy (specific IgE positivity to aeroallergens).

### Information on potential confounders

Smoking was recorded as: never smokers, ex-smokers, occasional smokers (<1 gram tobacco per day) and daily smokers. A history of chronic disease (yes/no) was defined as a positive answer to questions regarding physician diagnosed diabetes, hypertension, cerebrovascular disease, ischemic heart disease and other cardiac diseases. Social class was defined on the basis of questions regarding number of years of vocational training and employment status and categorised into five classes: class 1 (unemployed and no vocational training), class 2 (unemployed and >1 year of vocational training), class 3 (employed and no vocational training), class 4 (employed and 1–3 years of vocational training), class 5 (employed and >3 years of vocational training). Height and weight were measured wearing light clothes and no shoes. Body mass index (BMI) was calculated as weight divided by height squared. BMI were categorized according to the following criteria recommended by WHO: underweight (<18.5 kg/m^2^), normal range (≥18.5–25 kg/m^2^), overweight (≥25–30 kg/m^2^) and obese (≥30 kg/m^2^) [12].

### Genotyping of single nucleotide polymorphisms in the CHI3L1 gene

A region 22 kb upstream and 10 kb downstream of *CHI3L1* were chosen from the HapMap project (www.hapmap.org) and HapMap Data Rel 21a/phaseII Jan07, on NCBI assembly, dbSNP b125, were used for the SNP selection. A total of 11 tgSNPs (including rs10399931 and rs4950928) located 14 kb upstreams and 2 kb downstreams and covering all LD blocks in *CHI3L1* were genotyped. TAGGER chose these SNPs as the most informative in that +20 kb–−10 kb region.

TAGGER was used with a 5% MAF cut off and aggressive tagging, i.e. r^2^>0.8. Genotyping was performed using Taqman allelic discrimination (Kbioscience, Herts, UK) with a success rate >97.0%. Discordance was 0% as judged from re-genotyping of 965 random duplicate samples. Genotype distribution obeyed Hardy Weinberg Equilibrium (HWE), all p>0.25.

### Statistical analyses

Statistics were computed with the statistical program SAS, version 9.1 (SAS Institute Inc, Cary, NC, USA). All p-values reported are two-tailed and statistical significance was defined as p<0.05. All association analyses were done in the subgroup of never-smokers as well as in the total study population to see whether smoking amplified possible associations of SNPs in *CHI3L1* with asthma, atopy or asthma quantitative traits. The associations between SNPs in *CHI3L* and the dichotomous outcomes were examined by logistic regression. Results are reported as odds ratios (ORs) with 95% confidence intervals (95% CI). Differences were tested by the likelihood ratio test.

The association between *CHI3L1* SNPs and the continous outcomes were examined in linear regression models. Serum total IgE levels were log-transformed to achieve a normal distribution. Results are reported as β coefficients with 95% CI, except for serum total IgE levels where inverse transformations of model estimates were performed to provide proportional differences in % with 95% CI. Differences were tested by the F test. All regression models were adjusted for sex, age, smoking status (in the analyses of the total study population), BMI and social status. Age and BMI were tested for linear associations with the outcomes by including the squared terms of the variables in the respective models. In case of a non-linear relationship the variable was either categorized or the squared term of the variable was included in the model. Test for linear trends across genotype groups was performed by including the genotype groups as continuous variables in the regression models. P-values are not adjusted for multiple testing.

## Results

Clinical characteristics of the Inter99 cohort are presented in Table 1. A total of 2319 (35.9%) individuals were daily smokers. Atopy was diagnosed in 1994 (32.3%) individuals and physician-diagnosed was reported from 540 (8.6%) individuals. Atopic asthma was present in 300 (4.8%) individuals. In the subgroup of never-smokers (2202 (34.0%)), atopy was diagnosed in 781 (36.4%) individuals and physician-diagnosed was reported from 210 (9.5%) individulas. Atopic asthma was present in 145 (6.6%).

**Table 1:** Clinical characteristics of the study population.

Sex (males)	48.7 (3,169/6,514)^a^
Age	46.20 (30.63–61.72)^b^
Daily smokers	35.9 (2,319/6,467)^a^
Never-smokers	34.0 (2,202/6,467)^a^
BMI	26.3 (17.2–35.4)^b^
Highest social class (≥4 years education, employed)†	38.0 (2,282/6,002)^a^
Chronic disease††	22.2 (1,446/6,514)^a^
*“Has a physician ever told you that you have asthma?”*	8.6 (540/6,287)^a^
Atopy†††	32.3 (1,994/6,172)^a^
Atopic asthma	4.8 (300/6,270)^a^
Serum total IgE (kU/L)	29.85 (1.70–525.12)^b^
Percent of predicted FEV_1_	97.0 (68.8–125.2)^b^
Percent of predicted FVC	103.0 (75.2–130.9)^b^
FEV_1_/FVC in percent	78.8 (63.3–94.3)^b^

a % (n/ntotal).

b geometric mean (95% prediction interval).

BMI, body mass index.

† Social class were defined in 5 categories based on employment status and years of education.

†† A history of chronic disease was defined as a positive answer to questions regarding physician diagnosed diabetes, hypertension, cerebrovascular disease, ischemic heart disease, and other cardiac diseases.

††† Atopy was defined as a positive test for specific IgE to at least one of four common inhalant allergens.

The prevalences of the 11 tgSNPs of *CHI3L1* are shown in Table S1. All SNPs presented MAFs>5% except from rs4950930 (MAF = 4.5%).

Homozygosity of the rs4950928 G allele (MAF = 20.5%) as compared to homozygosity of the C allele was significantly associated with the prevalence of atopic asthma (OR 1.93 (95% CI, 1.21; 3.07) and with self-reported physician diagnosed asthma (OR 1.50 (95% CI, 1.00; 2.26) after adjustment for age, sex, smoking status, socio-economic class and BMI) (Table 2). In never-smokers similar adjusted association between homozygosity of the rs4950928 G allele and atopic asthma was seen (OR 2.17 (95% CI, 1.10; 4.29) (Table S2).

**Table 2:** Prevalence and risk (odds ratio (95% confidence interval))^a^ of atopy, atopic asthma and self-reported physician diagnosed asthma according to single nucleotide polymorphisms (SNPs) of *CHI3L1*.

SNP	Allele (major/minor)	Genotype	“Have a physician ever told you you had asthma?”		Atopic asthma‡		Atopy†	
			Prevalence	OR (95% CI)^c^	Prevalence	OR (95% CI)^c^	Prevalence	OR (95% CI)^c^
rs883125	C/G	CC	8.52 (366/4297)^a^	1.00	5.01 (215/4291)^a^	1.00	33.43 (1438/4302)^a^	1.00
		CG	9.07 (142/1565)	1.00 (0.80; 1.24)	4.66 (73/1565)	0.89 (0.67; 1.19)	29.86 (464/1554)	0.82 (0.72; 0.74)
		GG	6.62 (10/151)	0.69 (0.33;1.42)	3.97 (6/151)	0.60 (0.22;1.64)	29.93 (44/147)	0.86 (0.59; 1.26)
			p = 0.54^b^	p = 0.56	p = 0.75^b^	p = 0.44	p = 0.03^b^	p = 0.01
rs880633	C/T	CC	9.12 (162/1776)	1.00	4.85 (86/1773)	1.00	33.11 (587/1773)	1.00
		CT	8.15 (239/2934)	0.96 (0.77;1.20)	4.81 (141/2931)	1.03 (0.77; 1.38)	31.82 (933/2932)	0.95 (0.83; 1.09)
		TT	8.33 (105/1260)	1.00 (0.76;1.32)	4.84 (61/1260)	1.03 (0.72; 1.47)	32.51 (410/1261)	0.99 (0.84; 1.16)
			p = 0.50	p = 0.91	p = 1.00	p = 0.98	p = 0.65	p = 0.73
rs4950928	C/G	CC	8.94 (343/3838)	1.00	5.16 (198/3834)	1.00	31.84 (1218/3825)	1.00
		CG	7.40 (144/1947)	0.86 (0.69;1.06)	3.91 (76/1944)	0.76 (0.57; 1.01)	32.92 (643/1953)	1.07 (0.94; 1.20)
		GG	11.63 (30/258)	1.50 (1.00;2.26)	8.53 (22/258)	1.93 (1.21; 3.07)	36.72 (94/256)	1.28 (0.97; 1.68)
			p = 0.03	p = 0.04	p<0.01	p<0.01	p = 0.23	p = 0.17
rs10399931	C/T	CC	9.03 (317/3509)	1.00	5.22 (183/3506)	1.00	32.19 (1125/3495)	1.00
		CT	7.61 (162/1968)	0.87 (0.71;1.07)	4.14 (88/2128)	0.80 (0.61; 1.05)	32.41 (693/2138)	1.02 (0.90; 1.15)
		TT	10.43 (36/345)	1.30 (0.98;1.90)	6.67 (23/345)	1.39 (0.87; 2.21)	33.53 (115/343)	1.07 (0.84; 1.37)
			p = 0.08	p = 0.12	p = 0.06	p = 0.07	p = 0.88	p = 0.84
rs6691378	G/A	GG	8.67 (404/4662)	1.00	4.83 (225/4655)	1.00	32.70 (1525/4663)	1.00
		GA	8.12 (103/1268)	0.90 (0.71;1.15)	5.04 (64/1269)	0.96 (0.71; 1.31)	31.99 (404/1263)	0.96 (0.83; 1.10)
		AA	10.34 (9/87)	1.25 (0.59;2.64)	4.60 (4/87)	1.10 (0.39; 3.04)	30.59 (26/85)	0.86 (0.52; 1.43)
			p = 0.70	p = 0.57	p = 0.95	p = 0.96	p = 0.83	p = 0.70
rs4950930	G/A	GG	8.38 (462/5513)	1.00	4.74 (261/5506)	1.00	32.31 (1778/5503)	1.00
		GA	10.17 (49/482)	1.21 (0.86;1.69)	6.21 (30/483)	1.30 (0.86; 1.98)	33.81 (164/485)	1.05 (0.85; 1.29)
		AA	7.14 (1/14)	0.74 (0.10;5.76)	7.14 (1/14)	1.34 (0.17; 10.40)	28.57 (4/14)	0.76 (0.24, 2.47)
			p = 0.33	p = 0.54	p = 0.24	p = 0.48	p = 0.79	p = 0.82
rs12123883	T/C	TT	8.46 (439/5191)	1.00	4.76 (247/5191)	1.00	32.53 (1690/5195)	1.00
		TC	9.17 (74/807)	1.14 (0.86;1.49)	5.36 (43/802)	1.18 (0.83; 1.67)	30.95 (247/798)	0.92 (0.46; 1.85)
		CC	5.26 (2/38)	0.61 (0.14;2.57)	2.63 (1/38)	0.60 (0.08; 4.42)	33.33 (13.39)	0.92 (0.77; 1.09)
			p = 0.68	p = 0.50	p = 0.70	p = 0.55	p = 0.67	p = 0.59
rs2486064	G/A	GG	8.52 (174/2042)	1.00	4.84 (99/2044)	1.00	32.36 (655/2024)	1.00
		GA	8.39 (242/2885)	1.01 (0.81; 1.25)	4.65 (134/2883)	0.95 (0.72; 1.26)	32.05 (932/2908)	0.91 (0.85; 1.10)
		AA	9.37 (102/1089)	1.23 (0.94;1.61)	5.82 (63/1083)	1.31 (0.93; 1.84)	33.74 (363/1076)	1.07 (0.91; 1.27)
			p = 0.61	p = 0.25	p = 0.31	p = 0.15	p = 0.60	p = 0.42
rs2886117	G/A	GG	8.64 (399/4619)	1.00	4.81 (222/6119)	1.00	32.42 (1498/4620)	1.00
		GA	8.32 (110/1322)	0.92 (0.73;1.17)	5.14 (68/1324)	1.00 (0.74; 1.34)	32.50 (428/1317)	1.00 (0.87; 1.14)
		AA	8.41 (9/107)	0.97 (0.46;2.03)	4.67 (5/107)	1.11 (0.44; 2.76)	28.57 (30/105)	0.80 (0.51; 1.27)
			p = 0.93	p = 0.80	p = 0.89	p = 0.98	p = 0.70	p = 0.64
rs872129	A/G	AA	8.65 (442/5109)	1.00	4.94 (252/5103)	1.00	32.70 (1663/5086)	1.00
		AG	8.47 (74/874)	0.90 (0.68;1.19)	4.58 (40/874)	0.81 (0.55; 1.18)	30.47 (270/886)	0.87 (0.74; 1.03)
		GG	9.52 (4/42)	1.44 (0.50;4.13)	7.14 (3/42)	1.71 (0.52; 5.65)	35.71 (15/42)	1.05 (0.53; 2.08)
			p = 0.90	p = 0.59	p = 0.59	p = 0.37	p = 0.90	p = 0.27
rs871799	G/C	GG	8.58 (420/4896)	1.00	4.89 (239/4892)	1.00	32.46 (1583/4877)	1.00
		GC	8.47 (90/1062)	0.88 (0.68;1.14)	4.91 (52/1060)	0.84 (0.60; 1.18)	32.06 (345/1076)	0.94 (0.81; 1.09)
		CC	4.23 (3/71)	0.32 (0.08;1.33)	1.41 (1/71)	0.27 (0.04; 1.96)	36.23 (25/69)	1.04 (0.62; 1.75)
			p = 0.43	p = 0.12	p = 0.47	p = 0.17	p = 0.77	p = 0.68

† Atopy was defined as a positive test for specific IgE to at least one of four common inhalant allergens.

‡ Atopic asthma was defined as atopy in combination with self-reported physician-diagnosed asthma.

a % (n/n_total_). All such values. N_total_ may differ due to missing data.

b p values of chi square test/Fisher's exact test.

c OR (95% CI) were estimated in logistic regression models. Models were adjusted for sex, age, bmi, smoking status, and social class.

On the other hand CG heterozygothy of rs4950928 had a small protective effect against atopic asthma (OR 0.60 (95% CI; 0.39; 0.93) and physician diagnosed asthma (OR 0.64 (95% CI, 0.5; 0.91) among never-smokers when compared with CC homozygosity (Table S2).

In the total population but not in the subgroup of never-smokers, carriers of the G allele of rs883125 were protected against the prevalence of atopy (OR 0.82 (CI, 0.72; 0.94)) as compared to homozygosity of the C allele (Table 2).

In the total population homozygothy of the rs2486064 A allele was positively associated with shortness of breath when at rest (OR 1.33 (95% CI, 1.06; 1.68), p = 0.05) whereas homozygosity of the minor A allele of rs2886117 were protective against shortness of breath when at rest (OR 0.43 (95% CI, 0.18; 0.98), p = 0.02)(data not shown). Among never-smokers homozygosity of the rs2886117 A allele was found to be protective against atopy (OR 0.34 (95% CI, 0.13; 0.90) (Supplementary data, Table B). Neither rs2886117 nor rs2486064 were associated with any other clinical information of asthmatic symptoms, with the presence of atopic asthma or with measures of lung function neither in the total population nor among never-smokers (Table 2–3 and Table S2–S3).

**Table 3:** Lung function (mean (95%CI)) and effect (β coefficient (95% CI)) on lung function according to single nucleotide polymorphisms (SNPs) of *CHI3L1*.

SNP	Allele (major/minor)	Genotype	Percent of predicted FEV_1_		Percent of predicted FVC		FEV_1_/FVC in percent	
			Mean	Effect	Mean	Effect	Mean	Effect
rs883125	C/G	CC	96.9 (96.5; 97.3)	0.0	103.2 (102.7; 103.6)	0.0	78.7 (78.4; 78.9)	0.0
		CG	97.6 (96.8; 98.3)	0.7 (−0.1; 1.5)	103.1 (102.4; 103.8)	0.1 (−0.8; 0.9)	79.2 (78.8; 79.6)	0.5 (0.1; 1.0)
		GG	96.6 (94.3;98.9)	0.7 (−1.6; 3.0)	102.2 (99.8; 104.5)	0.2 (−2.1; 2.4)	78.7 (77.5; 80.0)	0.4 (−0.9; 1.7)
			p = 0.29; p_trend_ = 0.28	p = 0.21; p_trend_ = 0.09	p = 0.70; p_trend_ = 0.56	p = 0.99; p_trend_ = 0.87	p = 0.08; p_trend_ = 0.06	p = 0.07; p_trend_ = 0.04
rs880633	C/T	CC	97.0 (96.4; 97.7)	0.0	103.5 (102.8; 104.1)	0.0	78.6 (78.2; 79.0)	0.0
		CT	97.1 (96.6; 97.7)	0.0 (−0.8; 0.8)	103.1 (102.6; 103.6)	−0.3 (−1.2; 0.5)	78.9 (78.6; 79.2)	0.3 (−0.2; 0.7)
		TT	97.0 (96.2; 97.8)	−0.2 (−1.2; 0.9)	102.7 (101.9; 103.5)	−0.7 (−1.7; 0.3)	79.0 (78.5; 79.4)	0.4 (−0.2; 1.0)
			p = 0.94; p_trend_ = 0.99	p = 0.93; p_trend_ = 0.77	p = 0.38; p_trend_ = 0.16	p = 0.40; p_trend_ = 0.17	p = 0.42; p_trend_ = 0.21	p = 0.36; p_trend_ = 0.16
rs4950928	C/G	CC	97.1 (96.6; 97.5)	0.0	103.3 (102.9; 103.8)	0.0	78.7 (78.4; 78.9)	0.0
		CG	97.2 (96.6; 97.9)	0.1 (−0.7; 0.9)	103.0 (102.3; 103.6)	−0.3 (−1.0; 0.5)	79.0 (78.6; 79.3)	0.3 (−0.1; 0.7)
		GG	96.8 (94.9; 96.6)	−0.7 (−2.6; 1.1)	101.7 (99.9; 103.5)	−1.8 (−3.6; 0.0)	79.6 (78.6; 80.7)	0.8 (−0.2; 1.8)
			p = 0.88; p_trend_ = 0.92	p = 0.69; p_trend_ = 0.75	p = 0.18; p_trend_ = 0.10	p = 0.14; p_trend_ = 0.10	p = 0.11; p_trend_ = 0.04	p = 0.15; p_trend_ = 0.05
rs10399931	C/T	CC	97.2 (96.7; 97.7)	0.0	103.5 (103.0; 104.0)	0.0	78.7 (78.4; 78.9)	0.0
		CT	96.9 (96.3; 97.5)	−0.2 (−1.0; 0.6)	102.8 (102.1; 103.4)	−0.5 (−1.2; 0.3)	78.9 (78.6; 79.3)	0.2 (−0.2; 0.7)
		TT	97.5 (95.8; 99.3)	0.0 (−1.6; 1.6)	102.0 (100.4; 103.7)	−1.6 (−3.1; 0.0)	79.9 (79.0; 80.7)	1.1 (0.2; 2.0)
			p = 0.68; p_trend_ = 0.84	p = 0.87; p_trend_ = 0.74	p = 0.06; p_trend_ = 0.02	p = 0.11; p_trend_ = 0.04	p = 0.03; p_trend_ = 0.01	p = 0.04; p_trend_ = 0.02
rs6691378	G/A	GG	97.2 (96.7; 97.6)	0.0	103.2 (102.8; 103.6)	0.0	78.9 (78.7; 79.1)	0.0
		GA	96.8 (96.0; 97.6)	−0.4 (−1.3; 0.5)	102.9 (102.1; 103.7)	−0.2 (−1.0; 0.7)	78.6 (78.1; 79.1)	−0.2 (−0.7; 0.3)
		AA	96.5 (93.4; 99.6)	−1.1 (−4.2; 2.0)	102.1 (99.1; 105.1)	−1.3 (−4.4; 1.8)	79.1 (77.0; 81.1)	0.0 (−1.7; 1.8)
			p = 0.68; p_trend_ = 0.38	p = 0.52; p_trend_ = 0.26	p = 0.70; p_trend_ = 0.45	p = 0.68; p_trend_ = 0.49	p = 0.53; p_trend_ = 0.40	p = 0.69; p_trend_ = 0.47
rs4950930	G/A	GG	97.1 (96.7; 97.5)	0.0	103.1 (102.7; 103.5)	0.0	78.9 (78.7; 79.1)	0.0
		GA	96.9 (95.7; 98.2)	−0.3 (−1.6; 1.1)	103.6 (102.4; 104.8)	0.5 (−0.8; 1.8)	78.1 (77.4; 78.8)	−0.7 (−0.4; 0.1)
		AA	92.6 (82.6; 102.7)	−5.0 (−11.8; 1.8)	102.5 (94.1; 10.8)	−0.7 (−7.4; 6.1)	76.0 (71.1; 81.0)	−4.2 (−8.0; −0.4)
			p = 0.46; p_trend_ = 0.48	p = 0.33; p_trend_ = 0.38	p = 0.73; p_trend_ = 0.50	p = 0.73; p_trend_ = 0.52	p = 0.05; p_trend_ = 0.02	p = 0.02; p_trend_ = 0.02
rs12123883	T/C	TT	97.0 (96.6;97.4)	0.0	103.0 (102.6, 103.4)	0.0	78.8 (78.6; 79.1)	0.0
		TC	97.5 (96.5; 98.5)	0.50 (−0.6; 1.6)	103.8 (102.8; 104.7)	1.0 (−0.1; 2.0)	78.7 (78.1; 79.3)	−0.4 (−1.0; 0.2)
		CC	95.5 (90.1; 101.0)	0.50 (−4.0; 5.0)	100.4 (95.1; 105.7)	−0.9 (−5.4; 3.5)	79.8 (77.0; 82.5)	1.3 (−1.3; 3.8)
			p = 0.59; p_trend_ = 0.63	p = 0.64; p_trend_ = 0.36	p = 0.20; p_trend_ = 0.41	p = 0.17; p_trend_ = 0.14	p = 0.69_trend_ = 0.89	p = 0.24_trend_ = 0.42
rs2486064	G/A	GG	96.8 (96.2; 97.4)	0.0	102.8 (102.2; 103.4)	0.0	78.8 (78.5; 79.2)	0.0
		GA	97.4 (96.8; 97.9)	0.4 (−0.4; 1.2)	103.5 (102.9; 104.0)	0.6 (−0.2; 1.4)	78.8 (78.5; 79.1)	−0.2 (−0.6; 0.3
		AA	96.8 (96.9; 97.7)	0.0 (−1.1; 1.0)	102.8 (102.0; 103.6)	0.0 (−1.0; 1.0)	79.0 (78.5; 79.5)	0.0 (−0.6; 0.6)
			p = 0.32; p_trend_ = 0.70	p = 0.61; p_trend_ = 0.84	p = 0.22; p_trend_ = 0.71	p = 0.24; p_trend_ = 0.72	p = 0.76_trend_ = 0.67	p = 0.65_trend_ = 0.93
rs2886117	G/A	GG	97.2 (96.8; 97.6)	0.0	103.2 (102.8; 103.6)	0.0	78.9 (77.1; 80.5)	0.0
		GA	96.8 (96.9; 97.6)	−0.4 (−1.3; 0.4)	103.0 (102.2; 103.8)	−0.2 (−1.1; 0.7)	78.6 (78.1; 79.0)	−0.2 (−0.7; 0.3)
		AA	96.0 (93.2; 98.7)	−1.2 (−4.0; 1.6)	102.0 (99.2; 104.7)	−1.0 (−3.7; 1.8)	78.8 (77.1; 80.5)	−0.2 (−1.8; 1.3)
			p = 0.52; p_trend_ = 0.27	p = 0.44; p_trend_ = 0.21	p = 0.64; p_trend_ = 0.42	p = 0.73; p_trend_ = 0.45	p = 0.47; p_trend_ = 0.28	p = 0.65; p_trend_ = 0.37
rs872129	A/G	AA	97.1 (96.7; 97.5)	0.0	103.2 (102.8; 103.6)	0.0	78.8 (78.6; 79.0)	0.0
		AG	96.8 (95.9; 97.8)	−0.3 (−1.3; 0.8)	102.7 (101.8; 103.6)	0.0 (−0.9; 1.0)	79.0 (78.5; 79.5)	0.1 (−0.5; 0.7)
		GG	98.1 (93.2; 103.1)	1.0 (−3.5; 5.5)	102.4 (98.0; 106.9)	1.0 (−1.1; 3.1)	81.0 (78.7; 83.3)	1.5 (−1.0; 4.1)
			p = 0.78; p_trend_ = 0.75	p = 0.80; p_trend_ = 0.78	p = 0.65; p_trend_ = 0.36	p = 0.68; p_trend_ = 0.39	p = 0.17; p_trend_ = 0.17	p = 0.48; p_trend_ = 0.46
rs871799	G/C	GG	97.0 (96.6; 97.4)	0.0	103.1 (102.7; 103.5)	0.0	78.8 (78.6; 79.0)	0.0
		GC	97.4 (96.5; 98.3)	0.5 (−0.5; 1.4)	103.3 (102.4; 104.1)	0.3 (−0.6; 1.3)	78.9 (78.4; 79.4)	0.1 (−0.4; 0.6)
		CC	95.7 (92.1; 99.4)	−1.0 (−4.4; 2.4)	101.6 (98.0; 105.2)	−0.8 (−4.2; 2.6)	79.2 (77.2; 81.1)	−0.2 (−2.1; 1.7)
			p = 0.57; p_trend_ = 0.77	p = 0.51; p_trend_ = 0.56	p = 0.65; p_trend_ = 0.91	p = 0.70; p_trend_ = 0.70	p = 0.85; p_trend_ = 0.58	p = 0.92; p_trend_ = 0.83

Differences were estimated in linear regression models adjusted for sex, age, bmi, smoking status, and social class.

FEV_1_, forced expiratory volume in the first second; FVC, forced expiratory vital capacity.

In multivariate adjusted analyses none of the SNPs were significantly associated with FEV1 neither in the total population nor among never-smokers. In the total study population, both rs10399931 and rs4950930 were associated with FEV_1_/FVC-ratio (forced expiratory volume in the first second/forced expiratory vital capacity) (Table 3). Homozygosity of the minor T allele of rs10399931 had a positive effect on FEV_1_/FVC-ratio (β = 1.1 (95% CI, 0.2; 2.0)), whereas homozygosity of the minor A allele of rs4950930 had a negative effect on FEV_1_/FVC-ratio (β = −4.2 (95% CI, −8.0; −0.4)). Similar associations were not seen among never-smokers where homozygosity of the rs661378 A allele however was found to be associated with a lower FEV_1_/FVC ratio (β = 3.8 (95% CI, 0.9; 6.8) (Table S3). None of the remaining investigated variations of CHI3L1 were significantly associated with asthma, atopy or asthma-related traits or with measures of lung function (Table 3). None of the SNPs were associated with serum total IgE (data not shown).

A combined analysis (Mantel-Haenszel test) for asthma with our rs4950928 data and previously published rs4950928 data [6], [7] was performed. However, the three studies were significantly different and OR could not be combined in a meta-analysis (p = 0.01).

## Discussion

This is the first large-scale study of variation in *CHI3L1* in relation to asthma and atopy in a population-based sample of adults. *CHI3L1* encodes the inflammatory protein YKL-40 and circulating levels of this protein are reported to be elevated in patients with respiratory inflammatory diseases such as asthma [4], [6] and sarcoidosis [5], [13]. In patients with asthma, but not in patients with sarcoidosis, serum YKL-40 levels also seems to be a biomarker of severity of the disease [4]. Different genome wide association studies have shown, that SNPs in the *CHI3L1* promoter are associated with differential gene expression [14], transcript levels [15] and circulating YKL-40 levels [5], [14]. Recently, variation in the *CHI3L1* promoter was also found to associate with atopy [7].

We examined 6514 adult Danes of which 8.6% had physician diagnosed asthma, 4.8% suffered from atopic asthma and 32.3% were diagnosed with atopy. Similiar prevalences were found in the subgroup of never-smokers. In the total population we found an adjusted association between homozygosity of the rs4950928 G allele (minor allele) of *CHI3L1* and self-reported physician diagnosed asthma. This is in contradiction with a previous study of different populations by Ober et al [6]. Ober et al examined 4 different populations using the same diagnostic criterias and found an association between rs4950928 and asthma in 3 of 4 populations due to a higher prevalence of the C and not the G allele [6]. These 3 populations differed in many ways; one population consisted of 753 genetically related individuals [16] of which 63 suffered from asthma, another population consisted of 344 children with asthma (The Freiburg population) and the third population consisted of a mix of 99 children and adults with asthma recruited from an asthma clinic (Chicago population) [6]. Since the frequency of the G allele in our study is similar with MAF in these populations, the discrepancy between the two studies does not seem to arise from genotyping opposite strands. Smoking could be an influencing factor on this association, since we did not find similar association in the subgroup of never-smokers. However, among never-smokers we did not find any association between the rs4950928 C allele and asthma either. It has previously been shown, that rs4950928 also accounts for 9.4% of the variance in serum YKL-40 levels with the G allele having an additive negative effect on serum YKL-40 levels [6].

We also found homozygosity of the rs4950928 G allele to be positively associated with atopic asthma (defined as as a positive test for specific IgE to at least one of four common inhalant allergens combined with self-reported physician-diagnosed asthma) with the highest OR among never-smokers indicating that smoking does not enhance the OR. However, we found that rs4950928 CG heterozygosity was protective against physician diagnosed asthma and atopic asthma in the subgroup of never-smokers but not in the total population indicating that smoking obliterates this protective association. On the other hand, we also found that rs 883125 CG heterozygosity was associated with a significantly lower prevalence of atopy in the total population but not in the subgroup of never-smokers.

We did not find an association between rs4950928 and total serum IgE levels, which is in accordance with previous studies where rs4950928 was not associated with total serum IgE or atopy in any of the examined populations [6]. In a recent study of 295 Korean children with atopy, the rs10399805 *CHI3L1* promoter variant was associated with increased risk of atopy whereas an association between rs4950928 and atopy was not seen and in general no association between variations of *CHI3L1* and asthma was found [7].

Overall, we did not find convincing evidence that variations in *CHI3L1* have a strong influence on lung function, e.g. as reflected by predicted FEV1. Among never-smokers AA homozygosity of rs6691378 was associated with a higher FEV_1_/FVC-ratio which was not seen in the total population. However, two SNPs (rs10399931 and rs4950930) were associated with FEV_1_/FVC-ratio in the total population but not in the subgroup of never-smokers. In the present study, rs10399931 has a positive effect on FEV_1_/FVC-ratio which is in contrast with a previous study of patients with sarcoidosis [5]. Sarcoidosis patients have higher serum YKL-40 levels compared to healthy controls, and in sarcoidosis patients serum YKL-40 levels are correlated with carbon monoxide diffusing lung capacity (DL_CO_) but not with neither FEV_1_ nor FVC. However, only in the healthy controls, rs10399931 contributes to the interindividual variations of serum YKL-40 levels, and rs10399931 does not seem to influence sarcoidosis susceptibility or severity [5]. On the other hand, the negative effect of rs4950930 on FEV_1_/FVC-ratio has never been shown previously.

**Figure 1:**
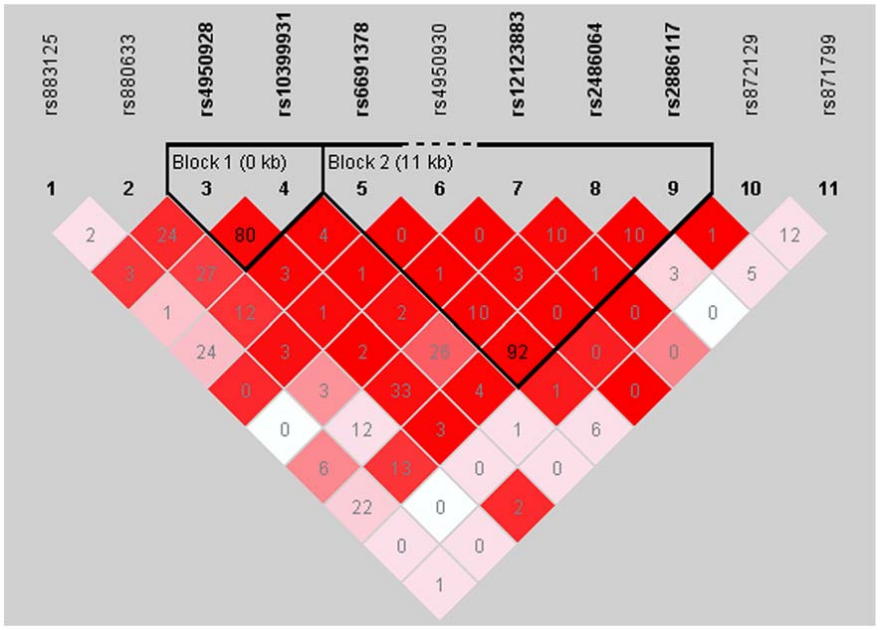
Linkage disequilibrium (LD) plot of the investigated variations of *CHI3L1.* Numbers represents the r^2^-values of the respective SNPs. The bolded fonts are representative of SNPs that are informative in the respective LD-block.

*CHI3L1* is located in an evolutionary conserved area of chromosome 1q32.1, and YKL-40 belongs to the family of 18-glycosyl hydrolases, which consists of chitinases as well as chitinase-like proteins. Chitinases have been shown to mediate airway inflammation [2] but even though YKL-40 lacks enzymatic properties, serum YKL-40 concentrations have been shown to be elevated in several acute and chronic inflammatory conditions including asthma [4], [6], [17]. YKL-40 is also known to participate in extracellular tissue remodelling and tissue fibrosis by increasing fibroblast cell proliferation [17], and several studies have shown, that YKL-40 plays a role in conditions leading to tissue fibrosis [17]–[20]. This is in accordance with the finding, that serum YKL-40 levels are elevated in patients with sarcoidosis, where it is expressed by macrophages both in the pulmonary sarcoid granulomas and in areas with inflammation [13]. A recent study shows, that YKL-40 is upregulated in patients with chronic obstructive pulmonary disease (COPD)[21], where it seems to contribute to tissue inflammation and remodelling by activating alveolar macrophages and sustaining their synthesis of proinflammatory and fibrogenic chemokines and metalloproteinases [21]. We did not find any associations between the examined SNPs of CHI3L1 and IgE levels, but a recent study in mice shows, that YKL-40 is induced during the pathogenesis of aero-allergen-induced Th2 inflammation and Il-13 effector responses and plays a critical role in allergen sensitization, IgE induction, Th2 cytokine production and macrophage activation [22]. All together, it seems that genetic variations in *CHI3L1* could account for inter-individual YKL-40 levels and that YKL-40 in asthma, sarcoidosis and COPD and plays a part of the pathogenesis with a role of acute inflammatory as well as chronic fibrotic character. A linkage disequilibrium (LD) plot of the examined variations of *CHI3L1* shows, that the two functional promoter SNPs of *CHI3L1*, rs4950928 and rs10399931, are in close LD (r^2^ = 0.80) (Figure 1). Pairwise these SNPs could account for a part of the role of *CHI3L1* in asthma; however the nominally associations of the individual SNPs does not add up to genotypes that are associated with both clinical informations and symptoms of asthma and/or atopy as well as measures of lung function.

In contrast to previous studies of variations in *CHI3L1* in relation to asthma, limitations of the present study are the lack of measurements of serum YKL-40. However, the criterias for the asthma diagnosis used in the present study are similar to the ones used in previous studies on the effects of variation in *CHI3L1* on asthma. The advantage of the present study is the size of the study population which adds statistical power and credibility to the outcome of the analyses. However, the associations are not corrected for multiple testing and some of the associations could be chance findings. Furthermore, the significant differences in prevalences of e.g. atopic asthma and effects on e.g. the FEV_1_FVC-ratio are rather small and probably without any clinical significance. Our results do not show, that smoking consistently influence the associations in an either positively og negatively way indicating that subgroup analyses correcting for smoking status compromise the statistical power of the analyses more than it strengthen the outcomes.

The meta-analysis of rs4950928 in asthma including our present study and the previous studies by Ober et al [6] and Sohn et al [7], showed significant heterogeneity and the three different studies could not be combined. This may be caused by the rather small sample sizes, and thus rather large confidence intervals from these studies, in the two previous studies showing association with asthma.

In conclusion, the present large population-based study investigated the associations of variations of *CHI3L1* with asthma and atopy in unrelated adults. Both in the total population and among never-smokers, The G allele of rs4950928 was associated with atopic asthma which is contradictory to previous findings. The rs4950928 G allele was also associated with self-reported physician diagnosed asthma in the total population. Among never-smokers rs4050928 CG heterozygosity was protective against atopic asthma and self-reported physcian-diagnosed asthma. Furthermore, in the total population carriers of the rs883125 G allele were found to be protected against atopy whereas homozygosity of the rs2886117 A allele was found to be protective against atopy among never-smokers. None of the SNPs were associated with FEV1, but two showed significant associations with the FEV_1_/FVC-ratio. Overall, the study does not consistently present specific variations of *CHI3L1* that are associated with both clinical informations and symptoms of asthma and/or atopy as well as measures of lung function but the study does not exclude a potential role of *CHI3L1* in susceptibility to asthma. Smoking does not consistently influence the associations in an either positively og negatively way. None of the significant associations in this report will resist corrections for multiple testing and obviously our findings are by nature explorative and they should be tested in independent study samples.

## Supporting Information

Table S1 (0.08 MB DOC)

Table S2 (0.12 MB DOC)

Table S3 (0.13 MB DOC)
