# Supplementary material for: Association of Polymorphisms of the CHI3L1 Gene with Asthma and Atopy: A Populations-Based Study of 6514 Danish Adults
Source: PLoS One. 2009 Jul 1;4(7):e6106. doi: 10.1371/journal.pone.0006106 (PMC2699472; doi:10.1371/journal.pone.0006106)
Supplement: Table S1 — (0.08 MB DOC) [file pone.0006106.s001.doc]

Table S1. Prevalence of the 11 single nucleotide polymorphisms (SNPs) of *CHI3L1*.

| **SNP** | **Allele** | **MAF** | **Genotype distribution** | |
| --- | --- | --- | --- | --- |
|  | (major/minor) | (%) | All | Prevalence, n (%) |
| rs883125 | C/G | 15.8 | CC | 4466 (71.7) |
|  |  |  | CG | 1607 (25.8) |
|  |  |  | GG | 156 (2.5) |
| rs880633 | C/T | 45.9 | CC | 1837 (29.7) |
|  |  |  | CT | 3041 (49.2) |
|  |  |  | TT | 1308 (21.1) |
| rs4950928 | C/G | 20.5 | CC | 3970 (63.4) |
|  |  |  | CG | 2025 (32.4) |
|  |  |  | GG | 265 (4.2) |
| rs10399931 | C/T | 23.9 | CC | 3628 (58.5) |
|  |  |  | CT | 2218 (35.8) |
|  |  |  | TT | 354 (5.7) |
| rs6691378 | G/A | 11.8 | GG | 4833 (77.5) |
|  |  |  | GA | 1314 (21.1) |
|  |  |  | AA | 88 (1.4) |
| rs4950930 | G/A | 4.5 | GG | 5713 (91.8) |
|  |  |  | GA | 498 (8.0) |
|  |  |  | AA | 15 (0.2) |
| rs12123883 | T/C | 7.7 | TT | 5387 (86.1) |
|  |  |  | TC | 827 (13.2) |
|  |  |  | CC | 40 (0.6) |
| rs2486064 | G/A | 42.4 | GG | 2108 (33.8) |
|  |  |  | GA | 3003 (48.2) |
|  |  |  | AA | 1122 (18.0) |
| rs2886117 | G/A | 13.0 | GG | 4788 (76.4) |
|  |  |  | GA | 1370 (21.9) |
|  |  |  | AA | 108 (1.7) |
| rs872129 | A/G | 8.4 | AA | 5285 (84.7) |
|  |  |  | AG | 912 (14.6) |
|  |  |  | GG | 43 (0.7) |
| rs871799 | G/C | 10.5 | GG | 5066 (81.1) |
|  |  |  | GC | 1111 (17.8) |
|  |  |  | CC | 71 (1.1) |

MAF, minor allele frequency.
