# Supplementary material for: Association of Polymorphisms of the CHI3L1 Gene with Asthma and Atopy: A Populations-Based Study of 6514 Danish Adults
Source: PLoS One. 2009 Jul 1;4(7):e6106. doi: 10.1371/journal.pone.0006106 (PMC2699472; doi:10.1371/journal.pone.0006106)
Supplement: Table S2 — (0.12 MB DOC) [file pone.0006106.s002.doc]

Table S2. Prevalence and risk (odds ratio (95% confidence interval))^a^ of atopy, atopic asthma and self-reported physician diagnosed asthma according to single nucleotide polymorphisms (SNPs) of *CHI3L* among never smokers.

| **SNP** | **Allele**  (major/minor) | **Genotype** | **”Have a physician ever told you**  **you had asthma?”** | | **Atopic asthma ‡** | | **Atopy †** | |
| --- | --- | --- | --- | --- | --- | --- | --- | --- |
|  |  |  | **Prevalence** | **OR (95% CI)^c^** | **Prevalence** | **OR (95% CI)^c^** | **Prevalence** | **OR (95% CI)^c^** |
| rs883125 | C/G | CC | 9.78 (148/1513) | 1 | 6.90 (104/1507) | 1 | 37.68 (564/1467) | 1 |
|  |  | CG | 8.96 (50/558) | 0.84 (0.59; 1.20) | 6.09 (34/558) | 0.83 (0.54; 1.27) | 34.48 (190/551) | 0.86 (0.69; 1.06) |
|  |  | GG | 8.70 (4/46) | 0.92 (0.32; 2.64) | 8.70 (4/46) | 1.38 (0.48; 3.97) | 31.82 (14/44) | 0.80 (0.42; 1.55) |
|  |  |  | p=0.84 | p=0.63 | p=0.70 | p=0.56 | p=0.33 | p=0.31 |
| rs880633 | C/T | CC | 10.58 (62/586) | 1 | 6.85 (40/584) | 1 | 38.95 (229/588) | 1 |
|  |  | CT | 8.65 (93/1075) | 0.83 (0.58; 1.20) | 6.15 (66/1073) | 0.91 (0.59; 1.41 | 36.11 (382/1058) | 0.93 (0.75; 1.16) |
|  |  | TT | 9.79 (43/439) | 1.00 (0.65; 1.54) | 7.55 (33/437) | 1.19 (0.72; 1.97) | 34.87 (151/433) | 0.91 (0.69; 1.19) |
|  |  |  | p=0.42 | p=0.51 | p=0.59 | p=0.53 | p=0.36 | p=0.75 |
| rs4950928 | C/G | CC | 10.63 (141/1327) | 1 | 7.41 (98/1323) | 1 | 36.49 (481/1318) | 1 |
|  |  | CG | 6.82 (49/718) | 0.64 (0.45; 0.91) | 4.61 (33/716) | 0.60 (0.39; 0.93) | 35.79 (253/707) | 0.99 (0.81; 1.21) |
|  |  | GG | 12.64 (11/87) | 1.40 (0.72; 2.74) | 12.64 (11/87) | 2.17 (1.10; 4.29) | 39.76 (33/83) | 1.34 0.84; 2.16) |
|  |  |  | p=0.01 | p=0.02 | p<0.01 | p<0.01 | p=0.77 | p=0.47 |
| rs10399931 | C/T | CC | 10.63 (129/1213) | 1 | 7.19 (87/1210) | 1 | 36.27 (437/1205) | 1 |
|  |  | CT | 7.87 (62/788) | 0.72 (0.52; 1.01) | 5.73 (45/786) | 0.78 (0.53; 1.16) | 36.71 (286/779) | 1.02 (0.84; 1.24) |
|  |  | TT | 9.17 (11/120) | 0.99 (0.51; 1.90) | 8.33 (10/120) | 1.40 (0.70; 2.81) | 36.21 (42/116) | 1.14 (0.76; 1.73) |
|  |  |  | p=0.12 | p=0.15 | p=0.34 | p=0.23 | p=0.98 | p=0.81 |
| rs6691378 | G/A | GG | 9.17 (151/1646) | 1 | 6.34 (104/1641) | 1 | 36.86 (600/1628) | 1 |
|  |  | GA | 10.55 (48/455) | 1.12 (0.78; 1.61) | 7.93 (36/454) | 1.20 (0.79; 1.82) | 36.89 (166/450) | 0.96 (0.77; 1.21) |
|  |  | AA | 4.00 (1/25) | 0.43 (0.06; 3.25) | 4.00 (1/25) | 0.63 (0.08; 4.78) | 20.00 (5/25) | 0.45 (0.16; 1.23) |
|  |  |  | p=0.44 | p=0.53 | p=0.42 | p=0.62 | p=0.22 | p=0.25 |
| rs4950930 | G/A | GG | 9.26 (179/1934) | 1 | 6.48 (125/1928) | 1 | 36.26 (693/1911) | 1 |
|  |  | GA | 10.71 (18/168) | 1.03 (0.59; 1.80) | 7.74 (13/168) | 0.97 (0.50; 1.89) | 40.00 (68/170) | 1.09 (0.78; 1.54) |
|  |  | AA | 0.00 (0/8) |  | 0.00 (0/8) |  | 25.00 (2/8) | 0.51 (0.10; 2.57) |
|  |  |  | p=0.54 | p=0.91 | p=0.62 | p=0.92 | p=0.50 | p=0.60 |
| rs12123883 | T/C | TT | 9.17 (168/1832) | 1 | 6.40 (117/1827) | 1 | 37.06 (673/1816) | 1 |
|  |  | TC | 9.96 (28/281) | 1.19 (0.77; 1.84) | 7.14 (20/280) | 1.23 (0.74; 2.05) | 31.88 (88/276) | 0.80 (0.60; 1.07) |
|  |  | CC | 25.00 (2/8) | 3.20 (0.62; 16.53) | 12.50 (1/8) | 2.01 (0.24; 16.80) | 37.50 (3/8) | 0.89 (0.21; 3.80) |
|  |  |  | p=0.29 | p=0.35 | p=0.71 | p=0.63 | p=0.25 | p=0.31 |
| rs2486064 | G/A | GG | 10.24 (38/371) | 1 | 6.11 (42/687) | 1 | 36.71 (250/681) | 1 |
|  |  | GA | 9.33 (99/1061) | 1.10 (0.77; 1.56) | 7.18 (76/1059) | 1.29 (0.85; 1.95) | 37.51 (395/1053) | 1.03 (0.83; 1.27) |
|  |  | AA | 9.45 (65/688) | 1.25 (0.80; 1.95) | 6.79 (25/368) | 1.25 (0.72; 2.15) | 33.88 (123/363) | 0.91 (0.69; 1.21) |
|  |  |  | p=0.87 | p=0.62 | p=0.69 | p=0.47 | p=0.46 | p=0.68 |
| rs2886117 | G/A | GG | 9.44 (153/1620) | 1 | 6.44 (104/1615) | 1 | 36.43 (585/1606) | 1 |
|  |  | GA | 10.17 (48/472) | 1.04 (0.73; 1.49) | 7.64 (36/471) | 1.13 (0.74; 1.71) | 38.28 (178/465) | 1.05 (0.84; 1.31) |
|  |  | AA | 6.25 (2/32) | 0.70 (0.16; 2.98) | 6.25 (2/32) | 1.03 (0.24; 4,42) | 15.63 (5/32) | 0.34 (0.13; 0.90) |
|  |  |  | p=0.73 | p=0.85 | p=0.65 | p=0.85 | p=0.04 | p=0.05 |
| rs872129 | A/G | AA | 9.49 (170/1791) | 1 | 6.61 (118/1786) | 1 | 36.92 (652/1766) | 1 |
|  |  | AG | 8.97 (28/312) | 0.84 (0.53; 1.34) | 6.43 (20/311) | 0.81 (0.46; 1.42) | 33.12 (104/314) | 0.79 (0.60; 1.04) |
|  |  | GG | 16.67 (3/18) | 2.61 (0.72; 9.43) | 16.67 (3/18) | 3.78 (1.04; 13.78) | 47.06 (8/17) | 1.46 (0.52; 4.11) |
|  |  |  | p=0.56 | p=0.30 | p=0.23 | p=0.15 | p=0.29 | p=0.18 |
| rs871799 | G/C | GG | 9.45 (162/1715) | 1 | 6.49 (111/1711) | 1 | 36.20 (614 (1996) | 1 |
|  |  | GC | 9.87 (37/375) | 0.94 (0.63; 1.42) | 7.77 (29/373) | 1.05 (0.66; 1.67) | 37.63 (140/372) | 0.99 (0.77; 1.28 |
|  |  | CC | 2.94 (1/34) | 0.32 (0.04; 2.40) | 2.94 (1/34) | 0.47 (0.06; 3.53) | 37.50 (12/32) | 0.90 (0.42; 1.92) |
|  |  |  | p=0.43 | p=0.41 | p=0.45 | p=0.69 | p=0.87 | p=0.96 |

† Atopy was defined as a positive test for specific IgE to at least one of four common inhalant allergens.

‡ Atopic asthma was defined as atopy in combination with self-reported physician-diagnosed asthma.

^a^ % (n/ n_total_). All such values. N_total_ may differ due to missing data.

^b^ p values of chi square test.

^c^ OR (95% CI) were estimated in logistic regression models. Models were adjusted for sex, age, bmi, and social class.
