# Supplementary material for: Association of Polymorphisms of the CHI3L1 Gene with Asthma and Atopy: A Populations-Based Study of 6514 Danish Adults
Source: PLoS One. 2009 Jul 1;4(7):e6106. doi: 10.1371/journal.pone.0006106 (PMC2699472; doi:10.1371/journal.pone.0006106)
Supplement: Table S3 — (0.13 MB DOC) [file pone.0006106.s003.doc]

Table S3. Lung function (mean (95%CI)) and effect (β coefficient (95% CI)) on lung function according to single nucleotide polymorphisms (SNPs) of *CHI3L1* among never smokers.

| **SNP** | **Allele**  (major/minor) | **Genotype** | **Percent of predicted FEV_1_** | | **Percent of predicted FVC** | | **FEV_1_/FVC in percent** | |
| --- | --- | --- | --- | --- | --- | --- | --- | --- |
|  |  |  | **Mean** | **Effect** | **Mean** | **Effect** | **Mean** | **Effect** |
| rs883125 | C/G | CC | 100.2 (99.6; 100.9) | 0 | 105.1 (104.4; 105.8) | 0 | 80.1 (79.8; 80.5) | 0 |
|  |  | CG | 100.8 (99.6; 101.9) | 0.4 (-0.8; 1.7) | 104.7 (103.5; 105.9) | -0.3 (-1.7; 1.0) | 80.8 (80.2; 81.5) | 0.7 (-0.1; 1.4) |
|  |  | GG | 99.2 (96.5; 101.9) | -1.0 (-4.9; 2.8) | 102.4 (99.0; 105.8) | -1.8 (-5.8; 2.2) | 81.1 (79.3; 83.0) | 0.6 (-1.6; 2.7) |
|  |  |  | p=0.59 | p=0.68 | p=0.37 | p=0.64 | p=0.10 | p=0.18 |
| rs880633 | C/T | CC | 100.6 (99.5; 101.6) | 0 | 105.8 (104.8; 106.8) | 0 | 80.2 (79.6; 80.8) | 0 |
|  |  | CT | 100.2 (99.4; 101.0) | -0.6 (-1.9; 0.7) | 104.8 (104.0; 105.6) | -0.9 (-2.3; 0.5) | 80.4 (79.9; 80.8) | 0.3 (-0.5; 1.0) |
|  |  | TT | 100.0 (98.7; 101.3) | -0.6 (-2.2; 1.1) | 104.5 (103.2; 105.8) | -0.8 (-2.5; 0.1) | 80.2 (79.6; 81.1) | 0.2 (-0.7; 1.1) |
|  |  |  | p=0.80 | p=0.65 | p=0.57 | p=0.43 | p=0.90 | p=0.76 |
| rs4950928 | C/G | CC | 100.4 (99.7; 101.1) | 0 | 105.1 (104.4; 105.8) | 0 | 80.3 (79.9;80.7) | 0 |
|  |  | CG | 100.1 (99.1; 101.1) | -0.4 (-1.6; 0.8) | 104.7 (103.6; 105.8) | -0.3 (-1.6; 1.0) | 80.3 (79.7; 80.8) | 0.0 (-0.7; 0.6) |
|  |  | GG | 100.7 (97.6;103.9) | 0.4 (-2.5; 3.3) | 104.1 (101.0; 107.3) | -0.9 (-3.9; 2.2) | 81.1 (79.4; 82.8) | 0.9 (-0.8; 2.5) |
|  |  |  | p=0.85 | p=0.76 | p=0.71 | p=0.80 | p=0.62 | p=0.56 |
| rs10399931 | C/T | CC | 100.4 (99.7; 101.2) | 0 | 105.3 (104.5; 106.0) | 0 | 80.2 (79.8; 80.6) | 0 |
|  |  | CT | 100.0 (99.0; 100.9) | -0.5 (-1.7; 0.7) | 104.4 (103.4; 105.4) | -0.6 (-1.8; 0.7) | 80.4 (79.9; 80.9) | 0.1 (-0.5; 0.8) |
|  |  | TT | 101.5 (98.9; 104.2) | 1.1 (-1.4; 3.6) | 105.0 (102.3; 107.7) | -0.4 (-3.0; 2.2) | 81.1 (79.8; 82.4) | 1.1 (-0.3; 2.5) |
|  |  |  | p=0.43 | p=0.42 | p=0.43 | p=0.65 | p=0.42 | p=0.32 |
| rs6691378 | G/A | GG | 100.6 (100.0; 101.2) | 0 | 105.2 (104.6; 105.9) | 0 | 80.4 (80.0; 80.7) | 0 |
|  |  | GA | 99.1 (97.9; 100.4) | -1.4 (-2.8; -0.1) | 103.8 (102.5; 105.1) | -1.1 (-2.5; 0.4) | 80.2 (79.4; 81.0) | -0.3 (-1.1; 0.5) |
|  |  | AA | 100.0 (95.7; 104.2) | -0.5 (-5.7; 4.8) | 99.5 (94.7; 104.2) | -5.2 (-10.7; 0.3) | 83.9 (81.5; 86.4) | 3.8 (0.9;b6.8) |
|  |  |  | p=0.10 | p=0.12 | p=0.02 | p=0.07 | p=0.05 | p=0.03 |
| rs4950930 | G/A | GG | 100.3 (99.7; 100.9) | 0 | 104.8 (104.2; 105.5) | 0 | 80.4 (80.1; 80.7) | 0 |
|  |  | GA | 100.9 (99.2; 102.6) | 0.9 (-1.2; 3.0) | 105.3 (103.4; 107.2) | 0.8 (-1.4; 3.0) | 80.2 (79.2; 81.1) | 0.0 (-1.2; 1.2) |
|  |  | AA | 95.1 (84.4; 105.9) | -3.8 (-12.0; 4.4) | 104.9 (96.8; 113.0) | 1.8 (-6.8; 10.4) | 76.3 (68.3;84.3) | -4.6 (-9.2; -0.1) |
|  |  |  | p=0.40 | p=0.45 | p=0.92 | p=0.71 | p=0.22 | p=0.14 |
| rs12123883 | T/C | TT | 100.2 (99.6; 100.8) | 0 | 104.7 (104.1; 105.4) | 0 | 80.4 (80.1; 80.7) | 0 |
|  |  | TC | 101.2 (99.6; 102.8) | 1.1 (-0.5; 2.8) | 106.3 (104.7; 107.9) | 1.5 (-0.2; 3.3) | 80.0 (79.1; 80.9) | -0.3 (-1.3; 0.6) |
|  |  | CC | 103.6 (95.4; 111.7) | 5.6 (-3.1; 14.3) | 107.6 (100.2; 115.0) | 4.9 (-4.2; 13.9) | 80.9 (74.9; 87.0) | 0.6 (-4.3; 5.4) |
|  |  |  | p=0.38 | p=0.19 | p=0.19 | p=0.14 | p=0.71 | p=0.76 |
| rs2486064 | G/A | GG | 99.9 (99.0; 100.8) | 0 | 104.4 (103.4; 105.4) | 0 | 80.5 (79.9;81.0) | 0 |
|  |  | GA | 100.8 (100.0; 101.5) | 0.5 (-0.7; 1.8) | 105.3 (104.5; 106.2) | 0.7 (-0.6; 2.0) | 80.3 (79.9; 80.7) | -0.1 (-0.9; 0.6) |
|  |  | AA | 99.7 (98.3; 101.1) | 0.0 (-1.2; 1.7) | 104.7 (103.2; 106.2) | 0.3 (-1.5; 2.0) | 80.1 (79.3; 80.9) | -0.2 (-1.1; 0.7) |
|  |  |  | p=0.28 | p=0.64 | p=0.37 | p=0.59 | p=0.77 | p=0.89 |
| rs2886117 | G/A | GG | 100.60 (99.98;101.23) | 0 | 105.21 (104.53;105.89) | 0 | 80.37 (80.03;80.71) | 0 |
|  |  | GA | 99.47 (98.25;100.69) | -1.13 (-2.47;0.21) | 104.15 (102.92;105.37) | -0.76 (-2.16;0.65) | 80.17 (79.41;80.92) | -0.33 (-1.08;0.43) |
|  |  | AA | 98.79 (94.84;102.75) | -1.87 (-6.46;2.71) | 100.56 (96.42;104.70) | -4.41 (-9.21;0.39) | 82.11 (79.78;84.45) | 1.93 (-0.64;4.51) |
|  |  |  | p=0.20 | p=0.20 | p=0.07 | p=0.13 | p=0.34 | p=0.22 |
| rs872129 | A/G | AA | 100.29 (99.69;100.90) | 0 | 104.99 (104.35;105.64) | 0 | 80.22 (79.88;80.57) | 0 |
|  |  | AG | 100.30 (98.83;101.76) | -0.36 (-1.96;1.23) | 104.37 (102.80;105.94) | -0.99 (-2.66;0.69) | 80.89 (80.16;81.61) | 0.50 (-0.40;1.40) |
|  |  | GG | 104.30 (97.43;111.16) | 4.83 (-1.75;11.40) | 106.19 (99.54;112.84) | 1.40 (-5.48;8.29) | 83.34 (81.09;85.59) | 2.90 (-0.79;6.60) |
|  |  |  | p=0.44 | p=0.31 | p=0.71 | p=0.46 | p=0.07 | p=0.18 |
| rs871799 | G/C | GG | 100.38 (99.76;100.99) | 0 | 105.02 (104.37;105.67) | 0 | 80.28 (79.93;80.63) | 0 |
|  |  | GC | 100.17 (98.85;101.49) | -0.20 (-1.67;1.28) | 104.74 (103.32;106.16) | -0.04 (-1.58;1.51) | 80.38 (79.68;81.08) | -0.13 (-0.96;0.70) |
|  |  | CC | 99.58 (95.17;103.98) | -0.36 (-4.81;4.08) | 102.27 (97.43;107.10) | -2.50 (-7.15;2.15) | 82.01 (79.53;84.50) | 1.87 (-0.63;4.37) |
|  |  |  | p=0.91 | p=0.96 | p=0.49 | p=0.57 | p=0.39 | p=0.32 |

Differences were estimated in linear regression models adjusted for sex, age, bmi, and social class.

FEV_1_, forced expiratory volume in the first second; FVC, forced expiratory vital capacity.
